# Toxicological Relevance of Pregabalin in Heroin Users: A Two-Year Postmortem Population Study

**DOI:** 10.1093/jat/bkab070

**Published:** 2021-06-10

**Authors:** Limon K Nahar, Kevin Murphy, Sue Paterson

**Affiliations:** Toxicology Unit, Imperial College London, Charing Cross Campus, St. Dunstan’s Road, London W6 8RP, UK; Department of Endocrinology and Metabolism, Imperial College London, Hammersmith Hospital, Du Cane Road, London, W12 0NN, UK; Toxicology Unit, Imperial College London, Charing Cross Campus, St. Dunstan’s Road, London W6 8RP, UK

## Abstract

Pregabalin (PGL) is a gabapentinoid used to treat epilepsy, neuropathic pain and generalized anxiety disorder. PGL is also misused by heroin users as it enhances the effects of heroin. While it is thought those who misuse PGL take it in amounts greater than the recommended therapeutic dose, it is unknown whether there is a significant difference between the amounts of PGL used by heroin users compared to non-heroin users. This study hypothesized that the PGL concentrations in postmortem (PM) samples taken from heroin users positive for PGL would be higher than those in non-heroin users. Between 1 January 2016 and 31 December 2016, a routine drug screen and a specific screen for PGL were carried out on femoral-vein bloods from 3,750 PM Coroners’ cases. Of the cases screened, 354 were heroin users, of which 264 cases were negative for gabapentinoids and therefore used as the control-heroin-user group. PGL was positive in 229 cases, of which 69 were heroin users and 160 were non-heroin users. On comparing the PGL concentrations, statistically higher concentrations were observed in the heroin users compared to non-heroin users (*P* = 0.002). There was no correlation between the concentrations of PGL and morphine (from heroin) in the heroin users (*P* = 0.95), and the amount of heroin (morphine) consumed was not dependant on whether PGL was consumed or not (*P* = 0.98). The prevalence of anti-depressants, benzodiazepines, methadone and non-heroin-related opioids was seen to be significantly higher in heroin users that were positive for PGL than the control-heroin users (*P* = < 0.001 for all drugs). This study suggests that heroin users are using greater amounts of PGL compared to non-heroin users; however, the magnitude of the difference in use may not be sufficient to conclude that heroin users are at substantially greater risk of PGL toxicity compared to non-heroin users. Results indicate that heroin users who take PGL are more likely to use multiple depressant drugs, hence increasing the risk of multi-drug toxicity and death in this population.

## Introduction

Pregabalin (PGL) has been prescribed in the UK since 2004 as Lyrica^®^ and is licensed to treat epilepsy, neuropathic pain and generalized anxiety disorder (1). PGL is a structural analog of gamma-aminobutyric acid (GABA) but does not directly interact with the GABA receptors (2). It is known to have a high affinity to α2δ-1 sub-units of voltage-sensitive Ca^2+^ channels, which decreases the release of excitatory neurotransmitters. This is thought to facilitate its analgesic, anxiolytic, anti-epileptic, sedative and muscle relaxant properties (3).

PGL was originally thought to have limited potential for misuse (4). However, it has been theorized that GABA analogs, such as PGL, can interact with the dopaminergic “reward” system, which is associated with drug addiction (5). Soon after its release, an illicit market for PGL started to develop both nationally and internationally (2, 6, 7). This was due to the ability of PGL to produce effects similar to those of traditional recreational drugs, including significant euphoric effects, improved sociability and relaxation (2).

Out of the drug-user population, heroin users and the prison population have the highest tendency for PGL abuse (2, 8–11). Heroin users report that PGL enhances the effects of heroin, while others report that using PGL helps them with their withdrawal symptoms and thus reduce their intake of heroin (12). However, no studies have been undertaken to establish if there is any relationship between the amount of PGL and heroin used by heroin users.

When heroin (diacetylmorphine) is ingested, it is rapidly metabolized to 6-acetylmorphine and then morphine. Due to the rapidity of heroin metabolism, generally, it is the morphine that is detected in biological matrices. Morphine is known to inhibit the gag reflex and to cause major respiratory and central nervous system (CNS) depression, which are the main causes of death among the heroin-user population (13). Similarly, PGL also has the propensity to cause respiratory and CNS depression (14), and studies have shown that concomitant use of opioids with PGL substantially increases the risk of opioid-related death compared to being treated with an opioid alone (15). Animal studies have suggested that PGL can reverse tolerance to morphine at low doses and that at higher doses it has an additive depressant effect on the respiratory system (12). Together, these reports suggest that misusing PGL with heroin increases the risk of toxicity and death by exacerbating depressive effects.

Many studies have reported a rise in PGL abuse (5, 16) and subsequent deaths. In England and Wales, registered deaths associated with PGL rose from 4 in 2012 to 187 in 2018 to 244 in 2019 (17). Registered deaths associated with heroin/morphine also rose from 579 in 2012 to 1,329 in 2019 (17). A previous study conducted by the authors on postmortem (PM) Coroners’ cases in England found the prevalence of PGL to be 4.1 times greater in heroin users than in those who did not use heroin (non-heroin users) (18). Evidence from patient interviews indicates that those who misuse PGL, such as heroin users, use it in amounts greater than the recommended therapeutic dose and that it is usually taken as a single dose (12, 19, 20). To date, no PM data have been published to determine whether those who misuse heroin with PGL are in fact consuming PGL at significantly higher dosage compared to those who do not use heroin, and whether this may therefore be contributing to heroin-associated deaths.

There are many PM toxicological studies describing PGL concentration ranges observed in PM blood. In a Finnish study in which all PM cases were screened for PGL in femoral-vein blood, the concentrations ranged from 0.28 to 110 µg/mL (*n* = 316), with 91% of those abusing PGL being positive for opioid (8). In a German study that screened for PGL in all PM cases, the PGL concentrations ranged from 0.04 to 23.8 µg/mL (median = 5.18 µg/mL, *n* = 43) with opioid being present in every PGL-positive case; morphine from heroin use was the third most common opioid detected (21). A UK study that screened for PGL selectively in PM cases (*n* = 93) found that the PGL concentrations detected ranged from <0.6 to 182 µg/mL, with ∼40% of the cases being positive for an opiate (codeine, heroin/morphine) and ∼65% positive for an opioid (22). An Australian study looking at coronial cases found PGL concentrations ranging from <0.05 to 140 mg/kg (median = 5.4 mg/kg, *n* = 209), with opioids being the most common additionally detected drugs (23). The concomitant use of opioids with PGL is mentioned in all reports as well as the use of heroin, but the PGL concentrations and corresponding morphine concentrations seen in the heroin users have not been described. To the best of the authors’ knowledge, there have been no studies to date describing the relationship between the concentrations of PGL and morphine seen in heroin users in PM blood samples.

In this study, the PGL femoral-vein blood concentrations from an entire PM cohort and defined subcategories, heroin users and non-heroin users, were evaluated. The distribution of PGL concentrations in heroin users was compared to non-heroin users to investigate whether there was a significant difference in the use of PGL between the groups. The correlation between the morphine from heroin use and corresponding PGL concentrations in the heroin users was investigated. The morphine concentrations, from heroin use, in heroin users that were positive for PGL were compared to the morphine concentrations in a control-heroin-user group (negative for gabapentinoids) to determine if the heroin users who used PGL consumed different amounts of heroin compared to those who did not use PGL. Concomitant detection of other drugs observed was also investigated between the groups. This study is a follow-up of a previous study conducted by the authors using the same PM population (18).

## Methods

The Toxicology Unit at Imperial College London performs toxicological analysis on PM samples submitted by pathologists on behalf of Coroners. Coroners’ jurisdictions covered by the Unit include seven of the eight jurisdictions in London and areas in the South East of England, covering ∼15% of England’s population.

Based on the request by the pathologist and consideration of the case history and samples submitted, PM analyses may include a routine toxicological drug screen of blood for prescribed, over-the-counter and controlled drugs using a basic liquid–liquid extraction (analysis by gas chromatography--mass spectrometry (GC--MS)) (24), measurement of alcohol (using headspace-GC with a flame ionization detector), urine drugs of abuse screen (solid-phase extraction (SPE) with GC--MS analysis) (25) and a specific screen for morphine (immunalysis—morphine-specific direct enzyme-linked immunosorbent assay [ELISA]). All morphine-positive blood samples are accurately quantified. All work is carried out in accordance with appropriate guidelines (26).

### Study population

Permission was obtained from the relevant Coroners to specifically screen for PGL on PM cases where femoral-vein blood was submitted from all deceased aged ≥16 years. This study commenced on 1 January 2016 and ended on 31 December 2017.

### Determining PGL concentrations

A simple protein precipitation method was used to screen/quantify PGL in femoral-vein blood. PGL and its deuterated analog (internal standard) were extracted from 100 μL of blood using a single addition acetonitrile protein precipitation reaction. Analysis was performed using liquid chromatography–tandem mass spectrometry. The assay was linear from 0.5 to 50.0 μg/mL. A PGL concentration of ≥ 0.5 µg/mL was recorded as positive finding. All positive samples were quantified accurately in duplicate (27).

### Determining morphine concentrations

In all cases that screened positive for morphine, the morphine was accurately quantified in duplicate using a fully validated in-house opioids method (includes morphine, 6-acetylmorphine and codeine). This method is a modification of a previously published method for buprenorphine in blood (28). Morphine and its internal standard, morphine-d_6_, were extracted from 1 mL of PM femoral-vein blood using cation exchange SPE cartridges (Bond Elut-Certify, Agilent Technologies, UK). Endogenous water-soluble compounds and lipids were removed from the cartridges using deionized water, followed by a solvent mix of hexane:propan-2-ol (1:2) and then methanol. Samples were then eluted using a solvent mix of chloroform:propan-2-ol:ammonia (80: 20: 2), concentrated under nitrogen and then derivatized using *N*-methyl-*N*-trimethylsilyltrifluoroacetamide. One microliter of each sample was then analyzed by GC--MS (Agilent Technologies, GC-68 90 N and MSD-5975) using an Rxi-5 MS column for chromatographic separation (Restek, 30 m × 0.25 mm I.D. × 0.25 mm). The initial oven temperature was set at 120°C that was raised to 310°C at the rate of 10°C/minute; the end temperature was held for 5 minutes giving a total run time of 24 minutes. The instrument was set to the electron impact-selective ion monitoring mode, monitoring *m/z* 429, 236, 196 and 435, 420, 421 for morphine and morphine-d_6_, respectively. The assay was linear from 0.01 to 1.00 µg/mL (*r^2^ >*0.99; *n* = 6) and intra-day (*n* = 6) and inter-day (*n* = 9) imprecision (%RSD) were less than 5% for morphine. Both limit of detection and quantification were set at 0.01 µg/mL. This method has been used routinely by the unit for over 10 years.

### Comparing PGL concentrations in heroin users to non-heroin users

To be placed in the “heroin-user” group, it was essential for the deceased to be positive for morphine in their blood or urine. The deceased also required an additional heroin-related compound (6-acetylmorphine, codeine from acetylcodeine, acetylcodeine, papaverine or noscapine) to be present or the case history had to strongly demonstrate heroin use (e.g., heroin or heroin-related paraphernalia at the scene, needle tracks, etc.). All cases that did not fall within the heroin-user group were categorized as “non-heroin users”.

To compare the concentrations of PGL in heroin users to non-heroin users, the cases that screened positive for PGL were subcategorized into heroin users and non-heroin users. The distribution of PGL concentrations was then compared between the two categories to determine if a significant difference was observed. Demographics and other toxicological findings were explored in both categories.

### Correlation between the morphine and PGL concentrations in heroin users

To determine if the amount of PGL used was related to the amount of heroin used, the PGL concentrations were plotted against the corresponding morphine concentrations (from heroin use) in the heroin users.

### Comparing morphine concentrations in heroin users that were positive for PGL to control-heroin users that were negative for gabapentinoids

A “control-heroin user” was defined as a heroin user who had screened negative for PGL and gabapentin. The distribution of morphine concentrations in the heroin users that were positive for PGL was compared to the morphine concentrations in the control heroin user group. Demographics and the concomitant detection of other drugs were also compared.

### Statistical analyses

All statistical analysis was carried out using IBM SPSS Statistics version 23.0 (Armonk, NY). Descriptive statistics were used to determine if the data were skewed or not, and parametric and non-parametric statistical analyses were used accordingly.

The Mann–Whitney U test was performed to test significance when the distribution was skewed. To determine if there was a correlation between the morphine and PGL concentration in heroin users, Spearman’s rank test (two-tailed) was used. A *P* value < 0.05 was considered significant. Pearson’s Chi-squared test (*χ*^2^ test) was applied to evaluate how likely it was that any observed differences in the prevalence of other drugs/drug groups between the heroin users positive for PGL and the control-heroin users arose by chance. An outlier concentration was defined as a concentration 1.5 times the interquartile range (IQR) of the concentrations distribution. An extreme outlier concentration was a concentration that was 3 (or more) times the IQR.

## Results

### Concentrations of PGL in femoral-vein blood

Between 1 January 2016 and 31 December 2017, the cohort consisted of 3,750 deceased. The cohort was predominantly male, containing 2,662 (71%) males and 1,088 (29%) females. 3,396 cases were non-heroin users, 354 were heroin users, of whom 264 cases were control-heroin users. Out of the 3750 cases screened, 229 were positive for PGL. The PGL concentrations in PM femoral-vein bloods ranged from 1 to 540 µg/mL (median = 6.0, ± 44.6 standard deviation (SD)). The Mann–Whitney U test showed no significant difference (*P* = 0.81) in the distribution of PGL concentrations between males (*n* = 154, range = 1–540 µg/mL, median = 6 µg/mL, ± 53.6 SD) and females (*n* = 75, range = 1–57 µg/mL, median = 7 µg/mL, ± 11.4 SD).

### Comparing PGL-positive heroin users to PGL-positive non-heroin users

#### Demographics

Of the 229 cases that screened positive for PGL, 69 (30%) were heroin users and 160 (70%) were non-heroin users. The proportion of male and female subjects, in both groups, were broadly consistent with the proportion for the whole cohort. Of the heroin users, 56 (81%) were male aged between 20 and 68 years (median = 44 years) and 13 (19%) were female aged between 23 and 53 years (median = 44 years). Of the non-heroin users (*n* = 160), 98 (61%) were male aged between 17 and 82 years (median = 45 years) and 62 (39%) were female aged between 16 and 91 years (median = 48 years). A two-tailed *t*-test showed no statistical difference between the age ranges for males and females, in the heroin users (*P* = 0.33) and the non-heroin users (*P* = 0.49).

#### Concomitant detection of other drugs

**Figure 1.**
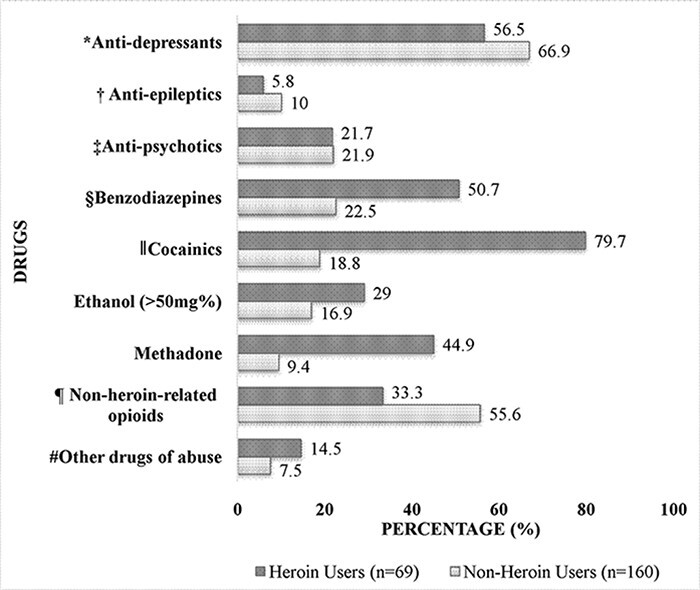
The concomitant use of other drugs with PGL in non-heroin users and heroin users. *Anti-depressants include but not limited to: Amitriptyline, citalopram, clomipramine, dothiepin, fluoxetine, mirtazapine, paroxetine, sertraline, trazodone, trimipramine, venlafaxine, etc. †Anti-epileptics include: Gabapentin, carbamazepine, clonazepam, lacosamide, lamotrigine, levetiracetam, phenytoin, valproate and zonisamidewere not screened for in all cases. ‡Anti-psychotics include: Clozapine, olanzapine, chlorpromazine and quetiapine. Aripiprazole and risperidone were not screened for in all cases. §Benzodiazepines include: Chlordiazepoxide, diazepam, desmethyldiazepam, temazepam, alprazolam. Alprazolam was not screened for in all cases. ║Cocainics include: Cocaine and/or cocaine metabolites in blood or urine. ¶Non-heroin-related opioids include: Medicinal morphine or morphine of unknown origin, codeine, dihydrocodeine, oxycodone, fentanyl and tramadol. Morphine and buprenorphine were not screened for in all cases. #Other Drugs of abuse include: Gamma-hydroxybutyric acid, cannabinoids, amfetamines, synthetic cannabinoids and designer benzodiazepines in blood or urine. These drugs were not screened for in all cases.

PGL was observed alone (no ethanol or other drugs) in only three cases (non-heroin users). In each of those cases, the PGL concentrations fell within therapeutic ranges seen in ante-mortem plasma (<14.1 µg/mL (29, 30)). In the heroin users (*n* = 69), the two most common drug groups observed were cocainics with 55 (79.7%) cases, followed by anti-depressants with 39 (56.5%) cases. The two most common drug groups observed in the non-heroin users (*n* = 160) were anti-depressants with 107 (66.9%) cases, followed by non-heroin-related opioids with 89 (55.6%) cases. Figure 1 presents the percentages of other drug groups detected with PGL in the heroin users and the non-heroin users.

#### Comparing PGL concentrations in heroin users to non-heroin users

**Figure 2.**
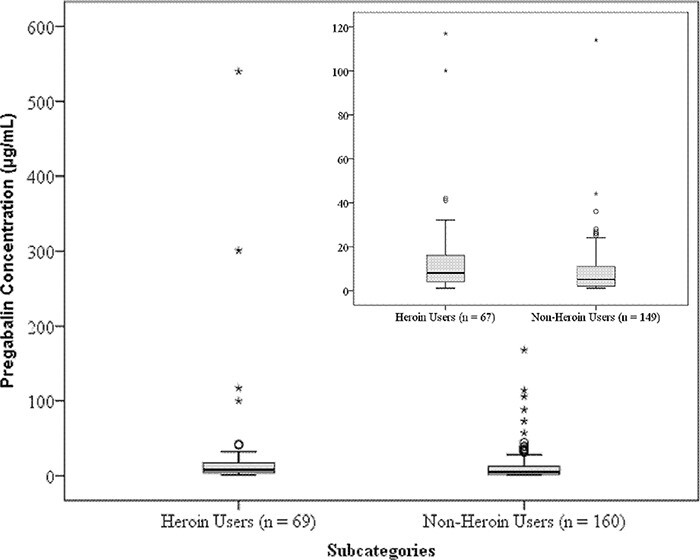
Distribution of PGL concentrations between heroin users and non-heroin users (PM femoral-vein blood) (all cases), with the inset displaying the boxplot excluding putative overdose cases. A circle (o) indicates an outlier value and an asterisk (*) indicates an extreme outlier value.

The PGL concentrations in the heroin users (*n* = 69) ranged from 1 to 540 µg/mL (median = 8.0 ± 74.2 SD) and from 1 to 168 µg/mL (median = 5.5 ± 21.0 SD) in the non-heroin users (*n* = 160) (see Figure 2). There was a statistically significant difference in the distribution of PGL concentrations between the groups, with higher concentrations of PGL being present in the heroin users, as analyzed by Mann–Whitney U test (*P* = 0.012).

Six outlier concentrations were present in the heroin users (41–540 µg/mL) and 14 in the non-heroin users (31–168 µg/mL) (see Figure 2). These outliers may include cases of intentional overdose of PGL, which may have skewed the results.

The case histories of the outliers were investigated to identify cases where there was a strong suggestion of intentional drug overdose, such as empty blister packets, suicide notes, tablet residues in cups at the scene, etc., or where the toxicology result showed extreme overdose concentrations of other drugs present. Two such cases were identified in the heroin-user category and 11 in the non-heroin users. These putative intentional overdose cases were removed from the dataset, and the remaining data were then reanalyzed. In the remaining cases, the PGL concentration ranged from 1 to 117 µg/mL (±19.3 SD) in the heroin users (*n* = 67), with the median concentration (50th percentile) at 8 µg/mL and 75th percentile at 16 µg/mL. In the non-heroin users (*n* = 149), the PGL concentrations ranged from 1 to 114 µg/mL (±11.7 SD) with the median concentration (50th percentile) at 5 µg/mL and 75th percentile at 11 µg/mL (see inset in Figure 2). The distribution of PGL concentrations remained statistically significantly higher in the heroin users (*P* = 0.002).

### Correlation between the morphine and PGL concentrations in heroin users (excluding putative intentional overdose cases)

Spearman’s rank test showed no statistically significant association between the PGL concentrations (range = 1–117 µg/mL) in the PM bloods of heroin users (*n* = 67) and the corresponding morphine concentrations from the same blood sample (range = 0.00–1.17 µg/mL, median = 0.14 µg/mL ± 0.26 SD) (*P* = 0.95). Nine PGL-positive heroin users were negative for morphine in the blood but positive for morphine and heroin-related compounds in the urine.

### Comparing PGL-positive heroin users to control-heroin users

#### Demographics

Of the heroin users that were positive for PGL (*n* = 69), 56 (81%) were male aged between 20 and 68 years, (median = 44 years) and 13 (19%) were female aged between 23 and 53 years (median = 44 years). Of the control-heroin users (*n* = 264), 223 (84%) were male aged between 16 and 71 years (median = 40 years) and 41 (16%) were female aged between 24 and 69 years (median = 48 years).

#### Comparing the morphine concentrations in the PGL-positive heroin users to the control-heroin users

There was no statistically significant difference in the distribution of morphine concentrations between PGL positive heroin users (*n* = 69, range = 0.00–1.46 µg/mL, median = 0.15 µg/mL) and the control-heroin users (*n* = 264, range = 0.00–20.00 µg/mL, median = 0.13 µg/mL ± 1.26 SD) (*P = *0.98 using a Mann–Whitney U test).

#### Comparing the concomitant detection of other drugs in PGL positive heroin users to control-heroin users

**Figure 3.**
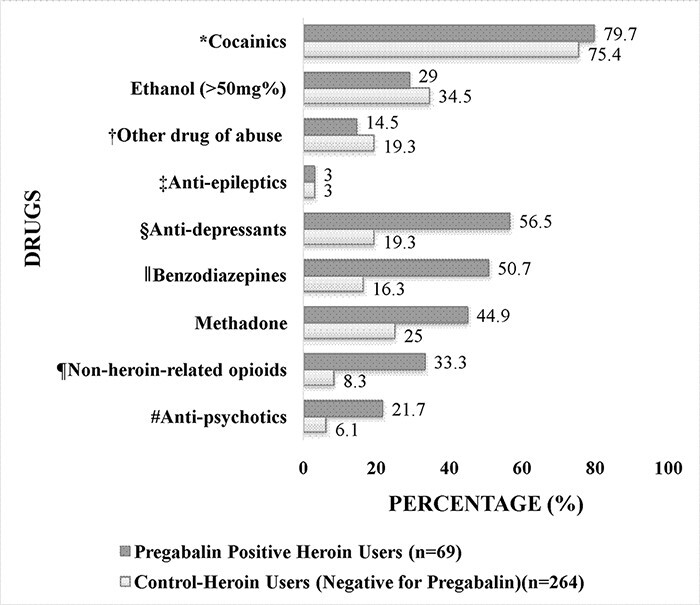
The concomitant use of other drugs in PGL positive heroin users and control-heroin users (negative for PGL). *Cocainics include: Cocaine and/or cocaine metabolites in blood or urine. †Other Drugs of abuse include: Gamma-hydroxybutyric acid, cannabinoids, amfetamines, synthetic cannabinoids and designer benzodiazepines in blood or urine. These drugs were not screened for in all cases. ‡Anti-epileptics include: Carbamazepine, clonazepam, lacosamide, lamotrigine, levetiracetam, phenytoin, valproate and zonisamidewere not screened for in all cases. Excludes gabapentin. §Anti-depressants include but not limited to: Amitriptyline, citalopram, clomipramine, dothiepin, fluoxetine, mirtazapine, paroxetine, sertraline, trazodone, trimipramine, venlafaxine, etc. ║Benzodiazepines include: Chlordiazepoxide, diazepam, desmethyldiazepam, temazepam, alprazolam. Alprazolam was not screened for in all cases. ¶Non-heroin-related opioids include: Medicinal morphine or morphine of unknown origin, codeine, dihydrocodeine, oxycodone, fentanyl, buprenorphine and tramadol. #Anti-psychotics include: Clozapine, olanzapine, chlorpromazine and quetiapine. Aripiprazole and risperidone were not screened for in all cases.

Cocainics (80 and 75%), ethanol of > 50 mg% (29 and 35%), other drugs of abuse (15 and 19%) and anti-epileptics (3 and 3%) were present at similar percentages in the heroin users positive for PGL (*n* = 69) and the control-heroin users (*n* = 264), respectively. However, the anti-depressants (57 and 19%), benzodiazepines (51 and 16%), methadone (45 and 25%), non-heroin-related opioids (33 and 8%) and anti-psychotics (22 and 6%) were present at higher percentages in heroin users positive for PGL compared to the control-heroin users (*P* ≤ 0.001 for all drugs by Chi-square test) (see Figure 3).

## Discussion and Conclusion

This is the first UK-based study that has screened for and quantified PGL in the femoral-vein bloods of an entire PM cohort (*n* = 3750). Our data show that heroin users who take PGL have a higher prevalence of prescription only CNS-depressant drugs present compared to those who take heroin without PGL. We also found that the prevalence of other CNS depressants is higher in non-heroin users who take PGL compared to heroin users positive for PGL. To the best of our knowledge, this is the first study to provide evidence that shows heroin users have statistically higher concentrations of PGL than non-heroin users (*P* = 0.002), albeit the difference observed may not be clinically significant.

The PGL concentrations seen ranged from 1 to 540 µg/mL (*n* = 229), a range comparable to those seen in PM bloods by other forensic laboratories, <0.1–226 µg/mL (includes femoral-vein, heart bloods, etc.) (8, 21, 22, 31). As PGL has a low volume of distribution (0.5–0.6 L/kg) (32), it undergoes minimum PM redistribution (as suggested by the limited information in the literature) (33); therefore, the PGL concentration observed in PM femoral-vein bloods is thought to reflect the concentration present in the deceased’s blood at the time of death.

On subcategorizing the PGL positive cohorts into heroin users (*n* = 69) and non-heroin users (*n* = 160), it was found that poly-pharmacy was common in both groups. The classic combination of drugs used by heroin users were as expected with cocainics, benzodiazepines and methadone being more prevalent in the heroin users compared to the non-heroin users. Interestingly, the results also showed that a high proportion of those who used PGL, in both heroin users and non-heroin users, were also positive for anti-depressants (56.5 and 66.9%) and non-heroin-related opioids (33.3 and 55.6%); both have CNS-depressive effects and will enhance the depressive effects with PGL. It needs to be highlighted that the prevalence of anti-depressants and non-heroin-related opioids was greater in the non-heroin users than the heroin users. These results show that those who take PGL are vulnerable individuals regardless of whether heroin is taken or not; they have the potential to take a mixed drug overdose either intentionally (due to mental health status) or accidently due to the combination of drugs that are available to them to take. Due to the findings of this study, the authors’ recommend that prescribers should be cautious when prescribing multiple CNS depressants to individuals. This is especially important when prescribing to those with a history drug misuse or mental health issues.

On comparing the distribution of PGL concentrations in heroin users (*n* = 67) to that in non-heroin users (*n* = 149), with the outliers removed, highly “significant” statistical differences were observed (*P *= 0.002). The 50th (median) and 75th percentiles of the PGL concentration distribution were 8 and 16 µg/mL in the heroin users and 5 and 11 µg/mL in the non-heroin users, respectively. Two clinical studies established that individuals given PGL at 600 mg per day (maximum recommended daily dose) had serum concentrations ranging from 0.87 to 14.2 µg/mL (29, 30). The median concentrations for PGL in both heroin users and non-heroin users were within the “therapeutic” range described in the literature, with slightly higher concentrations seen in the heroin users. The 75th percentile was marginally above the top limit of the “therapeutic” range in the heroin users and within the “therapeutic” range in the non-heroin users. Anecdotal evidence has suggested that those who misuse PGL use it at a greater dose than the recommended dose. This study is the first to report that heroin users are using statistically higher doses of PGL than non-heroin users; however, the difference observed may not be large enough to be clinically significant on its own (i.e., if morphine is not present in the blood of the heroin user). PGL is absorbed rapidly within an hour after oral ingestion, and its bioavailability is dose independent, with the maximum bioavailability remaining at ≥ 90% regardless of the dose (34). This pharmacokinetic property makes PGL dangerous, especially when combined with other CNS-depressant drugs. Studies have shown that the concomitant use of opioids with PGL substantially increases the risk of opioid-related death (15) and animal studies have suggested that PGL can reverse tolerance to morphine at low dosage (12). This means that the mere presence of PGL, even at low concentrations, in the presence of morphine (from heroin use) in the blood is an important finding as it may affect tolerance to morphine. Likewise, for non-heroin users who are prescribed or use opioids such as morphine and other CNS depressants with PGL would have similar increased risk of toxicity as the heroin users. The results from this study suggests that the heroin users are using higher doses of PGL than non-heroin users, but the magnitude of the difference is insufficient to conclude that heroin users are at substantially greater risk of PGL toxicity compared to non-heroin users. It may be possible that heroin users are simply dying sooner after consuming PGL with heroin than non-heroin users, and therefore, higher concentrations of PGL are being observed in the heroin users at PM analysis compared to a non-heroin user. Without knowing when a dose was taken in relation to time of death we are unable to establish this.

No correlation was observed between the concentrations of morphine from heroin use and PGL in the heroin users (*P* = 0.69). This suggests that there is no relationship between the amounts of heroin and PGL taken by an individual. No statistical difference was observed between the morphine concentrations (from heroin use) in the PGL-positive heroin users (*n* = 69) and the control-heroin users (*n* = 264). This indicates that the amount of heroin used is not dependant on whether or not PGL is taken (*P* = 0.98). It may be true that heroin users who state they are taking PGL to help with their withdrawal symptoms, are doing so, but are possibly taking PGL without reducing their intake of heroin. However, the lack of correlation seen may be due to the staggering of drug taking rather than taking PGL and heroin together and also because of the difference in the half-lives of both drugs (half-lives of heroin, morphine and PGL are 2–6 minutes, 1.3–6.7 hours and 5–9 hours, respectively) (32). Therefore, the correlation of the concentrations of morphine and PGL seen at PM may not correspond accurately with the actual dose taken prior to death. There are generally insufficient circumstantial details available regarding when or how the drugs were taken for the cases in the PM cohort. This is a well-known limitation of interpretation of PM case data.

When the concomitant use of other drugs was compared between the heroin users positive for PGL and the control-heroin users, unexpectedly, it was discovered that heroin users positive for PGL had significantly higher prevalence for anti-depressants (57 and 19%), benzodiazepines (51 and 16%), methadone (45 and 25%), non-heroin-related opioids (33 and 8%) and anti-psychotics (22 and 6%) compared to the control-heroin users. This high prevalence of benzodiazepines and methadone among those who also use PGL was also seen in a recent study from Israel that demonstrated that 31% of all patients on a methadone treatment program were misusing PGL, and all those who were misusing PGL were positive for benzodiazepines (35). The high prevalence of these CNS depressants in the heroin users that are positive for PGL strongly indicates that they are at a far greater risk of mixed-drugs toxicity than those who misuse heroin without PGL. This result may also suggest that the heroin users that are positive for PGL may be getting it prescribed to them as they are highly positive for other prescription only drugs (for pain management or other reasons.

If the latter is the case then these individuals require close monitoring by the prescribers.

Although a routine drugs screen was conducted on the entire PM cohort, not all cases underwent a specific morphine screen or urine drugs of abuse screen. This was due to a morphine screen not being requested by the pathologist or limitations of the specimens submitted for analysis. However, in all cases where the case documents mentioned any form of drug use or drug paraphernalia related to heroin use (opioid abuse, methadone, buprenorphine, naloxone, brown power, foil, etc.), a morphine screen was conducted on femoral-vein blood to ensure heroin use was not being missed. Due to the nature of PM analysis, only heroin users with relatively recent use of heroin prior to death could be included in the heroin-user category as the inclusion criteria required the presence of morphine in a biological specimen. This means that some individuals in the non-heroin-user category may be heroin users who had not used heroin recently. Unfortunately, full prescription histories and dosages are generally not provided with the coronial cases, which makes it difficult to determine if the PGL taken had been misused illicitly by the deceased or was a prescribed medication; such information would facilitate our understanding of drug taking patterns.

Our finding suggests that prescribers should to be cautions when co-prescribing CNS depressants with PGL to all patient groups not just to heroin users. It is recommended that all drug treatment centers should screen for illicit use of PGL on those who use heroin and those on the methadone maintenance program to help prevent PGL-associated heroin/opioid deaths.
